# Supplementary material for: Prospective, single UK centre, comparative study of the predictive values of contrast-enhanced ultrasound compared to time-resolved CT angiography in the detection and characterisation of endoleaks in high-risk patients undergoing endovascular aneurysm repair surveillance: a protocol
Source: BMJ Open. 2018 Apr 3;8(4):e020835. doi: 10.1136/bmjopen-2017-020835 (PMC5892749; doi:10.1136/bmjopen-2017-020835)
Supplement: Supplementary file 1 [file bmjopen-2017-020835supp001.pdf]

Data Collection Pro-forma

Participant Study ID:

Participant Questionnaire:

Reason for further investigation:

Height (cm):
Weight (kg):
Blood Pressure:
Pulse rate:

Functional Status:
0 - you are fully active, more or less as you were before your illness

1 - you cannot carry out heavy physical work, but can do anything else

2 - you are up and about more than half the day. You can look after yourself, but cannot work

Estimated walking distance:

Previous Heart Disease:
Known to have AF or other arythmia: Yes/No
Known IHD (Angina, ACS, MI): Yes/No   Details:…………………………………………
Previous Treatments:

Patients had echo in past: Yes/No   Details……………………………………..

Current Medications:

CDU:

Participant Study ID:

Completed by (investigators name):

Time start: Time Completed:

Diagnostic images obtained of:

Aortic neck	Yes/No		
Aneurysm body with graft in situ	Yes/No		
Bifurcation	Yes/No		
Right CIA (Midpoint of limb)	Yes/No	Left CIA (Midpoint of limb)	Yes/No
Right Limb/native transition	Yes/No	Left Limb/native transition	Yes/No

Aortic Measurements:

PSV in native aorta:

Native Aorta PI:

Neck / D2 diameter (mm):

Aneurysm /D3 diameter (mm):

Endoleak seen: Yes/No

Details:	Endoleak 1	Endoleak 2	Endoleak 3
Type:	Ia/Ib/II/III/Other	Ia/Ib/II/III/Other	Ia/Ib/II/III/Other
Inflow point(s):			
Outflow point(s):			
Certain of flow direction:	Yes/No	Yes/No	Yes/No

Limbs:

	Right	Left
Distal PSV measurement		
PI		
Wave form	Mono / Bi / Triphasic	Mono / Bi / Triphasic

Comments:

Common Femoral Arteries:

	Right	Left
Distal PSV measurement		
PI		
Wave form	Mono / Bi / Triphasic	Mono / Bi / Triphasic

Comments:

CEUS:

Participant Study ID:
Completed by (investigators name):
Time start: Time Completed:

First Contrast Injection: (all timings from start of contrast injection)

Time till seen in graft(s):
Time till seen in endoleak (s):

Contrast seen in Endoleak 1:	Yes / No	Endoleak 1 Type: Ia/Ib/II/III/Other
Contrast Seen in Endoleak 2:	Yes / No	Endoleak 2 Type: Ia/Ib/II/III/Other
Contrast seen in Endoleak 3:	Yes / No	Endoleak 3 Type: Ia/Ib/II/III/Other

Second Contrast Injection:

Time till seen in graft(s):
Time till seen in endoleak (s):

Contrast seen in Endoleak 1:	Yes / No	Endoleak 1 Type: Ia/Ib/II/III/Other
Contrast Seen in Endoleak 2:	Yes / No	Endoleak 2 Type: Ia/Ib/II/III/Other
Contrast seen in Endoleak 3:	Yes / No	Endoleak 3 Type: Ia/Ib/II/III/Other

Temporal CTA (in scan measurements)

Participant Study ID: Completed by (investigators name):

Time start: Time Completed:

Temporal CTA (reporting)

Completed by (investigators name):

Time reporting started: Time reporting finished:

Phase 2.5 Seconds

Image quality:	Fully diagnostic	5	4	3	2	1	Non-diagnostic

Endoleak seen: Yes/No

Details:	Endoleak 1	Endoleak 2	Endoleak 3
Type:	Ia/Ib/II/III/Other	Ia/Ib/II/III/Other	Ia/Ib/II/III/Other
Inflow point(s):			
HU at inflow:			
Outflow point(s):			
HU at outflow:			
Certain of flow direction:	Yes/No	Yes/No	Yes/No

Hounsfield Unit measurements: (excluding any calcification of graft structures)

Inferior vena cava: .... HU

Aortic lumen at superior fabric markers of endograft: .... HU

Aortic lumen at bifurcation of endograft: .... HU

Iliac lumen at Distal end of right limb of endograft .... HU

Iliac lumen at distal end of left limb of endograft .... HU

Phase 5 Seconds

Image quality:	Fully diagnostic	5	4	3	2	1	Non-diagnostic

Endoleak seen: Yes/No

Details:	Endoleak 1	Endoleak 2	Endoleak 3
Type:	Ia/Ib/II/III/Other	Ia/Ib/II/III/Other	Ia/Ib/II/III/Other
Inflow point(s):			
HU at inflow:			
Outflow point(s):			
HU at outflow:			
Certain of flow direction:	Yes/No	Yes/No	Yes/No

Hounsfield Unit measurements: (excluding any calcification of graft structures)

Inferior vena cava: .... HU

Aortic lumen at superior fabric markers of endograft: .... HU

Aortic lumen at bifurcation of endograft: .... HU

Iliac lumen at Distal end of right limb of endograft .... HU

Iliac lumen at distal end of left limb of endograft .... HU

Phase 7.5 Seconds

Image quality:	Fully diagnostic	5	4	3	2	1	Non-diagnostic

Endoleak seen: Yes/No

Details:	Endoleak 1	Endoleak 2	Endoleak 3
Type:	Ia/Ib/II/III/Other	Ia/Ib/II/III/Other	Ia/Ib/II/III/Other
Inflow point(s):			
HU at inflow:			
Outflow point(s):			
HU at outflow:			
Certain of flow direction:	Yes/No	Yes/No	Yes/No

Hounsfield Unit measurements: (excluding any calcification of graft structures)

Inferior vena cava: ………………. HU
Aortic lumen at superior fabric markers of endograft: ………………. HU
Aortic lumen at bifurcation of endograft: ………………. HU
Iliac lumen at Distal end of right limb of endograft ………………. HU
Iliac lumen at distal end of left limb of endograft ………………. HU

Phase 10 Seconds

Image quality:	Fully diagnostic	5	4	3	2	1	Non-diagnostic

Endoleak seen: Yes/No

Details:	Endoleak 1	Endoleak 2	Endoleak 3
Type:	Ia/Ib/II/III/Other	Ia/Ib/II/III/Other	Ia/Ib/II/III/Other
Inflow point(s):			
HU at inflow:			
Outflow point(s):			
HU at outflow:			
Certain of flow direction:	Yes/No	Yes/No	Yes/No

Hounsfield Unit measurements: (excluding any calcification of graft structures)

Inferior vena cava: ………………. HU
Aortic lumen at superior fabric markers of endograft: ………………. HU
Aortic lumen at bifurcation of endograft: ………………. HU
Iliac lumen at Distal end of right limb of endograft ………………. HU
Iliac lumen at distal end of left limb of endograft ………………. HU

Phase 15 Seconds

Image quality:	Fully diagnostic	5	4	3	2	1	Non-diagnostic

Endoleak seen: Yes/No

Details:	Endoleak 1	Endoleak 2	Endoleak 3
Type:	Ia/Ib/II/III/Other	Ia/Ib/II/III/Other	Ia/Ib/II/III/Other
Inflow point(s):			
HU at inflow:			
Outflow point(s):			
HU at outflow:			
Certain of flow direction:	Yes/No	Yes/No	Yes/No

Hounsfield Unit measurements: (excluding any calcification of graft structures)

Inferior vena cava: ………………. HU
Aortic lumen at superior fabric markers of endograft: ………………. HU
Aortic lumen at bifurcation of endograft: ………………. HU
Iliac lumen at Distal end of right limb of endograft ………………. HU
Iliac lumen at distal end of left limb of endograft ………………. HU

Phase 20 Seconds

Image quality:	Fully diagnostic	5	4	3	2	1	Non-diagnostic

Endoleak seen: Yes/No

Details:	Endoleak 1	Endoleak 2	Endoleak 3
Type:	Ia/Ib/II/III/Other	Ia/Ib/II/III/Other	Ia/Ib/II/III/Other
Inflow point(s):			
HU at inflow:			
Outflow point(s):			
HU at outflow:			
Certain of flow direction:	Yes/No	Yes/No	Yes/No

Hounsfield Unit measurements: (excluding any calcification of graft structures)

Inferior vena cava: ………………. HU
Aortic lumen at superior fabric markers of endograft: ………………. HU
Aortic lumen at bifurcation of endograft: ………………. HU
Iliac lumen at Distal end of right limb of endograft ………………. HU
Iliac lumen at distal end of left limb of endograft ………………. HU

Phase 25 Seconds

Image quality:	Fully diagnostic	5	4	3	2	1	Non-diagnostic

Endoleak seen: Yes/No

Details:	Endoleak 1	Endoleak 2	Endoleak 3
Type:	Ia/Ib/II/III/Other	Ia/Ib/II/III/Other	Ia/Ib/II/III/Other
Inflow point(s):			
HU at inflow:			
Outflow point(s):			
HU at outflow:			
Certain of flow direction:	Yes/No	Yes/No	Yes/No

Hounsfield Unit measurements: (excluding any calcification of graft structures)

Inferior vena cava: ………………. HU
Aortic lumen at superior fabric markers of endograft: ………………. HU
Aortic lumen at bifurcation of endograft: ………………. HU
Iliac lumen at Distal end of right limb of endograft ………………. HU
Iliac lumen at distal end of left limb of endograft ………………. HU

Venous Phase

Image quality:	Fully diagnostic	5	4	3	2	1	Non-diagnostic

Endoleak seen: Yes/No

Details:	Endoleak 1	Endoleak 2	Endoleak 3
Type:	Ia/Ib/II/III/Other	Ia/Ib/II/III/Other	Ia/Ib/II/III/Other
Inflow point(s):			
HU at inflow:			
Outflow point(s):			
HU at outflow:			
Certain of flow direction:	Yes/No	Yes/No	Yes/No

Hounsfield Unit measurements: (excluding any calcification of graft structures)

Inferior vena cava: ………………. HU
Aortic lumen at superior fabric markers of endograft: ………………. HU
Aortic lumen at bifurcation of endograft: ………………. HU
Iliac lumen at Distal end of right limb of endograft ………………. HU
Iliac lumen at distal end of left limb of endograft ………………. HU

Other comments / diagnostic findings:
