# Supplementary material for: Prospective, single UK centre, comparative study of the predictive values of contrast-enhanced ultrasound compared to time-resolved CT angiography in the detection and characterisation of endoleaks in high-risk patients undergoing endovascular aneurysm repair surveillance: a protocol
Source: BMJ Open. 2018 Apr 3;8(4):e020835. doi: 10.1136/bmjopen-2017-020835 (PMC5892749; doi:10.1136/bmjopen-2017-020835)
Supplement: Author's manuscript [file bmjopen-2017-020835.draft_revisions.pdf]

BMJ Open

BMJ Open is committed to open peer review. As part of this commitment we make the peer review history of every article we publish publicly available.

When an article is published we post the peer reviewers' comments and the authors' responses online. We also post the versions of the paper that were used during peer review. These are the versions that the peer review comments apply to.

The versions of the paper that follow are the versions that were submitted during the peer review process. They are not the versions of record or the final published versions. They should not be cited or distributed as the published version of this manuscript.

BMJ Open is an open access journal and the full, final, typeset and author-corrected version of record of the manuscript is available on our site with no access controls, subscription charges or pay-per-view fees (http://bmjopen.bmj.com).

If you have any questions on BMJ Open's open peer review process please email
info.bmjopen@bmj.com

BMJ Open

Protocol: A Prospective Single Centre Study of the Predictive Values of Contrast Enhanced Ultrasound compared to Time-Resolved Computer Tomography Angiography in the Detection and Characterisation of Endoleaks in High Risk EVAR Surveillance Patients

Journal:	*BMJ Open*
Manuscript ID	bmjopen-2017-020835
Article Type:	Protocol
Date Submitted by the Author:	27-Nov-2017

Complete List of Authors:	Roy, Iain; University of Liverpool Institute of Ageing and Chronic Disease; Royal Liverpool University Hospital, Liverpool Vascular & Endovascular Service<br>Chan , Tze; Royal Liverpool University Hospital, Department of Radiology<br>Czanner, Gabriela; University of Liverpool Institute of Ageing and Chronic Disease; University of Liverpool, Department of Biostatistics<br>Wallace, Stven; Royal Liverpool University Hospital, Liverpool Vascular & Endovascular Service<br>Vallabhaneni, Srinivasa; University of Liverpool Institute of Ageing and Chronic Disease; Royal Liverpool University Hospital, Liverpool Vascular & Endovascular Service

Keywords:	Ultrasound < RADIOLOGY & IMAGING, VASCULAR SURGERY, Cardiovascular imaging < RADIOLOGY & IMAGING, Computed tomography < RADIOLOGY & IMAGING, Aneurysm, Diagnostic radiology < RADIOLOGY & IMAGING

SCHOLARONE™
Manuscripts

Full Title:

Protocol: A Prospective Single Centre Study of the Predictive Values of Contrast Enhanced Ultrasound compared to Time-Resolved Computer Tomography Angiography in the Detection and Characterisation of Endoleaks in High Risk EVAR Surveillance Patients

Short Title: Contrast Enhanced Ultrasound, True Endoleak Detection rate

Authors:

Iain N Roy MBChB MRCS[1,2], Tze Yuan Chan MBChB FRCR[3], Gabriela Czanner PhD[1,4], Steve Wallace BSc AVS[2], Srinivasa Rao Vallabhaneni MD, FRCS[1,2].

1. Institute of Ageing & Chronic Disease, University of Liverpool, UK
2. Liverpool Vascular & Endovascular Service, Royal Liverpool University Hospital, UK
3. Department of Radiology, Royal Liverpool University Hospital, UK
4. Department of Biostatistics, University of Liverpool, UK

Correspondence:

Mr Iain Roy

Liverpool Vascular & Endovascular Service (LiVES)

Royal Liverpool Hospital

Prescot Street

Liverpool

L7 8XP

Telephone: +44 151 706 3457

Fax : +44 151 706 5827

E-mail: iain.roy@liverpool.ac.uk

Article Word Count: 3002 words

Journal Subject Codes: Diagnostic Testing, Imaging, Ultrasound, Aneurysm

For peer review only

Abstract

Introduction

Diagnosis of endoleaks is imperative to prevent failure of endovascular aneurysm repairs (EVAR). The gold standard for diagnosis of endoleaks is catheter directed subtraction angiography, which is not a practicable choice for surveillance. Computer Tomography Angiography (CTA) is the historical surveillance modality of choice. Concerns over cost, potential nephrotoxicity of contrast agents and repeated radiation exposure led to Colour Duplex Ultrasound Scan (CDUS) becoming an established alternative. CDUS has a lower sensitivity and specificity for endoleaks detection compared to CTA. Contrast Enhanced Ultrasound Scan (CEUS) represents an improvement of ultrasound imaging but comparisons against CTA report widely varying results, likely due to technical factors of CEUS and limitations of single phase CTA.

The development of time-resolved CTA (tCTA) offers timing information that much more closely mirrors the dynamic information available from CEUS. Theoretically these two imaging modalities have the best potential for diagnostic accuracy. The aim of this study will be to compare CEUS to tCTA and investigate the utility of other measurements available from tCTA.

Methods and Analysis

This is a prospective, single centre, comparative study of paired binary diagnostic imaging modalities. Patients identified in routine post EVAR surveillance as at risk of having a graft-related endoleak will undergo a CEUS and time-resolved CTA (tCTA) on the same day. This will allow the first comparison of CEUS to a semi-dynamic form of CTA. CEUS sensitivity and specificity to endoleak detection will be calculated.

Ethics and dissemination

The study has achieved ethical approval. We hope the results will define the diagnostic accuracy of CEUS in comparison to a semi-dynamic form of CTA, representing a methodological improvement

For peer review only

from previous studies. Results will be submitted for presentation at national and international Vascular Surgery & Radiology meetings. The full results are planned to be published in a medical journal.

Trial registration

ClinicalTrials.gov: NCT02688751

For peer review only

Strengths and limitations of this study

Strengths

- First comparison of Contrast Enhanced Ultrasound to a dynamic form of CT imaging, representing a methodological improvement
- Both imaging modalities occur on the same day removing changing findings as a confounding factor.
- Primary outcome set as Type I/III endoleak (most clinically significant) rather than all endoleaks

Limitations

- Appropriately powered but small study
- Single Centre study

For peer review only

Introduction

Background and rationale

Endovascular aneurysm repair (EVAR) is the intervention of choice to treat abdominal aortic aneurysms (AAA).[1] In comparison to open surgical repair (OSR), EVAR confers a reduction of mortality lasting into the short to intermediate term.[2] However, EVAR is associated with complications which sometimes require secondary interventions in order to maintain efficacy of EVAR. This has been recognised since the inception of the technique and confirmed in observational studies as well as RCT s.[3-6] Therefore periodic surveillance imaging is recommended for life following EVAR.[7 8] The importance of post-EVAR surveillance remains enduring, its value further highlighted by a recently published analysis of 15-year follow-up after EVAR[2] .

The commonest complication in EVAR surveillance is an endoleak,[9] which is "persistent blood flow within the aneurysm sac but outside the stent-graft".[10] Endoleaks are classified based on the source of blood flow ,[10] but can be grouped into stent-graft related (Types I and III) and type II (non stent-graft related) endoleaks. Stent-graft related endoleaks generally transmit high pressure causing a high risk of aneurysm expansion/rupture (treatment failure)[11 12]. In contrast, type II endoleaks generally run a benign course, particularly in the absence of aneurysm expansion.[12] With regards to endoleak imaging, high sensitivity of detection and high specificity of characterisation improve diagnostic utility of surveillance, in particular with an emphasis on distinguishing stent-graft related endoleaks from type II endoleaks. Digital subtraction angiography in multiple planes and a high frame rate of acquisition is the gold standard of endoleak imaging; high frame rates to demonstrate endoleak heamodynamics for better characterisation. However, this modality is not tenable to be used as surveillance imaging.

Historically EVAR surveillance was undertaken using Computer Tomography Angiography (CTA). Arterial phase CT was the most frequently used modality, although selectively or routinely additional phases are used such as un-enhanced, venous phase and even delayed phase. Concerns over cost,

use of potentially nephrotoxic contrast agent and repeated radiation exposure led to alternative imaging modalities being investigated and implemented in surveillance regimens. Colour Duplex Ultrasound Scan (CDUS) is the most widely used imaging modality currently.[13] CDUS is reported to have a lower sensitivity and specificity to detect stent-graft related endoleaks compared to CTA. [14]

Contrast Enhanced Ultrasound Scan (CEUS) has been investigated as an adjunct to CDUS in the hope of improving sensitivity to endoleak detection. CEUS involves intravenous injection of a microbubble contrast which remains in the blood, allowing improved detection of endoleaks, particularly with contrast coherent ultrasound imaging. CEUS also allows continuous (dynamic) or real time monitoring of the aneurysm and endoleak as the contrast agent arrives into the endoleak. Modern microbubble agents are expired by the respiratory system, thus avoiding nephrotoxicity. A recent review of 30,222 administrations of a CEUS contrast agent demonstrated a low adverse reaction rate of 0.020%.[15] CEUS also obviates the radiation exposure associated with CTA.

Systematic review of the diagnostic accuracy of CEUS (in comparison to CTA) in detection of any endoleak revealed variable sensitivity ranging from 67- 100% and specificity ranging from 79-100%.[16] The value of this review is compromised by heterogeneity of contrast material used and non-reporting of ultrasound imaging technique used, specifically whether colour duplex combined with contrast or harmonic CEUS imaging was used. There are potentially additional reasons variability in the reporting such as operator dependence, quality of equipment used as well as body habitus of the patient. The consideration that neither CEUS nor single phase CTA represent gold standard of endoleak diagnosis led to the metanalysis[14 16] adopting a bivariate model of analysis. This approach does not compensate for the lack of gold standard comparator as it favours any modality producing false positives. Comparison against the gold standard has never been established for either modality.

Time-resolved CT

For peer review only

Time-resolved CTA (tCTA) was first described for endoleak detection in 2010[17]. The single arterial phase is replaced by multiple phases in tCTA, which are typically of lower radiation dose, thus offering dynamic observations of endoleaks, such as flow direction and filling speed, while still retaining many of the advantages of CTA (3D reconstruction etc.) which closely mirror the advantages of the Multi-planar Digital subtraction catheter angiography. The multiple phases of tCTA can be achieved by broad CT detectors and a static patient in the Fowler position[18] or rapid shuttling of a supine patient through a standard detector[19 20]. Sufficient amount of measurements regarding filling patterns of endoleaks on tCTA[20] are now available to be able to replace a standard arterial phase in CTA with a tCTA that is aimed at detecting stent-graft related endoleaks, without increasing radiation exposure for the patient. Now timings and interpretation of tCTA are understood it is timely to do a comparison of CEUS to the improved comparator of tCTA. This overcomes the limitations of previous studies by comparison of CEUS to a (semi) dynamic form of CTA imaging as a gold standard.

Methods

Study Design

This is a prospective single centre comparative study of paired diagnostic imaging modalities, designed to comply with the "Standards for Reporting Diagnostic accuracy studies".[21 22] Participants will be recruited from a city-wide vascular service. The service is arranged in a hub and spoke configuration locally and regularly accepts tertiary referrals for complications of previous aortic surgery at other centres. EVAR surveillance is predominately undertaken using CDUS and plain radiography.[23]

Participants

Potential participants are referred to the study by their vascular surgeon when they require a CTA for further investigations of an endoleak following routine EVAR surveillance and meet inclusion criteria (TABLE 1). They are then assessed by the study for eligibility based on exclusion criteria

(TABLE 2) and approached to participate in the study, if appropriate. Patients are approached by an investigator and those who give their written informed consent to participate will be enrolled in the study.

Test Methods

Participants attend and have a CEUS (index test) and tCTA (reference standard) on the same day. This represents a change from standard care for participants who would otherwise have a CEUS and triple phase CTA (non-contrast, arterial (20s) and delayed venous phase (90s)) often on separate days. Participants will also be asked a short number of closed questions to assess functional status and cardiac function (supplementary material). Upon completion of the CEUS & tCTA, participants are returned to their referring surgeons, with clinical reports of the studies for ongoing care. The study team follows the participants further treatment and investigation until the end of the study.

Contrast Enhanced Ultrasound (CEUS)

Contrast enhanced ultrasound is performed in combination with a standard Colour Duplex Ultrasound Scan in our institution. It is reported as a binary test yielding two values: present or absent for each endoleak type. It is conducted by an experienced Clinical Vascular Scientist with extensive involvement in scans for EVAR surveillance. It is performed on a Philips IU22 ultrasound machine (Philips, Amsterdam, Netherlands), using the a 2-5MHz abdominal curved array probe. Grey scale images of the aneurysm neck (when possible), iliac seal zones and maximum aneurysm dimensions are obtained and measured in maximum antero-posterior and medio-lateral dimensions. Note is made of the echogenicity of thrombus within the aneurysm sac. Using colour flow imaging and spectral Doppler, waveform characteristics and velocities are recorded in the common femoral arteries. The stent-graft is interrogated using colour and spectral Doppler to ascertain patency and flow heamodynamics of the neck, main body as well as both limbs. Any abnormalities in these parameters are reported. Colour Doppler is used to detect any endoleak. If present, its type, point of inflow, point of outflow and flow dynamics (using sectoral doppler) are reported.

For peer review only

Optimum views of the area of concern are obtained, prior to contrast injection, using appropriate machine set ups and controls as determined by the operator. 2.4mls Sulphur Hexafluoride Microbubble Contrast (SonoVue™, Bracco, Milan, Italy) is injected followed by 10mls of sodium chloride 0.9%, the on-screen timer is started at the start of the injection. Flow direction and filling time ideally should be determined and anatomy of the endoleak established by interrogation. Passive elimination of the contrast agent is allowed to occur and the process repeated for a second injection.

CEUS scan will be reported by the performing vascular scientist to the data point recorded in the data collection proforma (supplementary material), in addition to any clinically relevant points. The vascular scientist will be blinded to the concurrent tCTA at the time of reporting, although will be aware of the previous findings on EVAR surveillance.

Time-resolved Computer Tomography Angiography (tCTA)

tCTA is performed on a Siemens Definition AS+ scanner (Seimens, Munich, Germany), in our institution. Participants are positioned supine with arms raised above their head. The contrast injector is connected to a 20G (or larger) IV catheter in an anterior cubital fossa vein. A standard topogram scan is performed. Unless not required, a non-contrast scan is performed. The maximum length that can be covered for the time resolution required is 27cm. This is centred over the EVAR stent-graft. Abdominal guides are placed at upper aspect of diaphragm and common femoral arteries for venous phase of scan. Abdominal aorta, just proximal to EVAR graft is selected as trigger area for time-resolved phases.

Contrast is injected, using an auto injector, at 4ml/s for 96mls. Participants are asked to adopt shallow breaths and not hold their breath. The time-resolved phase is triggered by a HU>90 in trigger area. Phases occur at 2.5, 5, 7.5, 10, 15, 20 and 25 seconds following the automatic trigger, these occur in cranio-caudually acquisition, except the 5 & 7.5 second phases which occur caudo-

For peer review only

cranially direction. The venous phase is taken in full inspiration and is acquired 75s following the trigger. Tube setting and calculated predicted radiation exposure are presented in Table 3.

All tCTA scans will be reported by a single consultant vascular radiologist. This reporter will be blinded to the results of the CEUS and collect data to the proforma. It will be reported as a binary test yielding two values: present or absent for each type of endoleak.

Outcome Measures

The primary outcome is:

The predictive values of CEUS in comparison to tCTA (as comparator) to detect stent-graft related endoleaks.

Secondary outcomes are:

1. Any adverse events during CEUS or tCTA.
2. Predictive values of CEUS in comparison to tCTA to detect type II endoleaks
3. Predictive values of both tCTA & CEUS in predicting final endoleak diagnosis (following any further investigations).
4. Predictive values of both tCTA & CEUS in predicting need for a secondary intervention.
5. Evaluate the association between CEUS temporal delay (difference between contrast in endograft & contrast in endoleak) and evaluate its ability to improve the differentiation of endoleak type.
6. Evaluate the association between "CEUS contrast in endoleak" to "tCTA contrast in endoleak" and assess potential as predictive tool, for optimum timing of CTA phases.

Analysis Plan

Associations will be established / refuted with summary statistics and graphical analysis and appropriate further statistical testing within the framework of logistic regression. If association can be established then predictive modelling will be undertaken. The agreement between CEUS and

For peer review only

tCTA will be evaluated with Kappa statistic.[24] Sensitivity/specificity will be calculated along with binomial exact 95% confidence intervals and leave-one-out cross-validation. We will also report the positive predictive value and negative predictive value.

The power calculation,[25] showed the required sample size to be 74. This was calculated based on a prevalence of stent-graft related endoleaks of 11% as demonstrated on previous tCTA studies of endoleaks. It was powered to detect a predicted sensitivity of 0.95 with a tolerated confidence interval of ±0.15. The study commenced recruitment in February 2016 and is on-going.

Monitoring & Data

All patients referred to the study are recorded in the screening log. The sponsoring trust will provide governance oversight and annual reports to the REC will provide ethical overview. Radiation exposure of participants will be monitored and reported to the sponsors and approving research ethics committee. No interim analysis is planned.

Upon completion of the study, all identifiable patient demographics will be removed and anonymised data will be stored to allow for future analysis of unforeseen benefits. During the study, data is stored in a secure manner within the hosts institutions data management processes. Access is restricted to the investigators and for audit by the sponsor.

Ethics and dissemination

The main ethical consideration is the change of care from CTA to a tCTA, this was felt to be appropriate in the context of informed consent, now it can be performed without increasing radiation exposure. Ethical approval (15/NW/0908) was granted by a NHS Research Ethics Committee. Upon completion of analysis we expect to publish in a medical journal, along with the anonymised data set and present the findings widely.

For peer review only

Authors' contributions

IR – Developed and wrote the protocol

TC – Reviewed and revised the radiological/imaging elements of the protocol

SW – Reviewed and revised the ultrasound elements of the protocol

GC – Reviewed and revised the statistical analysis elements of the protocol

SV – Conceived the project and supervised IR in developing the protocol

Funding statement

This research received no specific grant from any funding agency in the public, commercial or not-for-profit sectors.

It is sponsored by: Royal Liverpool & Broadgreen University Hospitals Hospitals NHS Trust (Ref: 5083) and supported by use of staff and resources from The Royal Liverpool University Hospital, Liverpool, UK and The Institute of Ageing & Chronic Disease, The University of Liverpool, UK.

Competing interest's statement

None

For peer review only

References

1. Waton S JA, Heikkila K, Cromwell D, Loftus I. National Vascular Registry: 2016 Annual report. London: The Royal College of Surgeons of England, November 2016.
2. Patel R, Sweeting MJ, Powell JT, et al. Endovascular versus open repair of abdominal aortic aneurysm in 15-years' follow-up of the UK endovascular aneurysm repair trial 1 (EVAR trial 1): a randomised controlled trial. *The Lancet*;388(10058):2366-74. doi: 10.1016/S0140-6736(16)31135-7
3. Blankensteijn JD, de Jong SECA, Prinssen M, et al. Two-Year Outcomes after Conventional or Endovascular Repair of Abdominal Aortic Aneurysms. *New England Journal of Medicine* 2005;352(23):2398-405. doi: 10.1056/NEJMoa051255
4. Becquemin JP, Pillet Jc Fau - Lescalie F, Lescalie F Fau - Sapoval M, et al. A randomized controlled trial of endovascular aneurysm repair versus open surgery for abdominal aortic aneurysms in low- to moderate-risk patients. (1097-6809 (Electronic))
5. Lederle FA, Freischlag JA, Kyriakides TC, et al. Long-Term Comparison of Endovascular and Open Repair of Abdominal Aortic Aneurysm. *New England Journal of Medicine* 2012;367(21):1988-97. doi: 10.1056/NEJMoa1207481
6. Investigators TUKET. Endovascular versus Open Repair of Abdominal Aortic Aneurysm. *New England Journal of Medicine* 2010;362(20):1863-71. doi: 10.1056/NEJMoa0909305
7. Moll FL, Powell JT, Fraedrich G, et al. Management of abdominal aortic aneurysms clinical practice guidelines of the European society for vascular surgery. *Eur J Vasc Endovasc Surg* 2011;41 Suppl 1:S1-S58. doi: 10.1016/j.ejvs.2010.09.011
8. Chaikof EL, Brewster DC, Dalman RL, et al. SVS practice guidelines for the care of patients with an abdominal aortic aneurysm: executive summary. *J Vasc Surg* 2009;50(4):880-96. doi: 10.1016/j.jvs.2009.07.001
9. Hobo R, Buth J, collaborators E. Secondary interventions following endovascular abdominal aortic aneurysm repair using current endografts. A EUROSTAR report. *J Vasc Surg* 2006;43(5):896-902. doi: 10.1016/j.jvs.2006.01.010
10. White GH, May J, Waugh RC, et al. Type III and type IV endoleak: toward a complete definition of blood flow in the sac after endoluminal AAA repair. *J Endovasc Surg* 1998;5(4):305-9. doi: 10.1583/1074-6218(1998)005<0305:TIATIE>2.0.CO;2
11. Veith FJ, Baum RA, Ohki T, et al. Nature and significance of endoleaks and endotension: summary of opinions expressed at an international conference. *J Vasc Surg* 2002;35(5):1029-35.
12. Harris PL, Vallabhaneni SR, Desgranges P, et al. Incidence and risk factors of late rupture, conversion, and death after endovascular repair of infrarenal aortic aneurysms: The EUROSTAR experience. *Journal of Vascular Surgery*;32(4):739-49. doi: 10.1067/mva.2000.109990
13. Karthikesalingam A, Page AA, Pettengell C, et al. Heterogeneity in surveillance after endovascular aneurysm repair in the UK. *Eur J Vasc Endovasc Surg* 2011;42(5):585-90. doi: 10.1016/j.ejvs.2011.06.053
14. Karthikesalingam A, Al-Jundi W, Jackson D, et al. Systematic review and meta-analysis of duplex ultrasonography, contrast-enhanced ultrasonography or computed tomography for surveillance after endovascular aneurysm repair. *The British journal of surgery* 2012;99(11):1514-23. doi: 10.1002/bjs.8873 [published Online First: 2012/09/25]
15. Tang C, Fang K, Guo Y, et al. Safety of Sulfur Hexafluoride Microbubbles in Sonography of Abdominal and Superficial Organs: Retrospective Analysis of 30,222 Cases. *Journal of ultrasound in medicine : official journal of the American Institute of Ultrasound in Medicine* 2017;36(3):531-38. doi: 10.7863/ultra.15.11075
16. Abraha I, Luchetta ML, De Florio R, et al. Ultrasonography for endoleak detection after endoluminal abdominal aortic aneurysm repair. *Cochrane Database Syst Rev* 2017;6:CD010296. doi: 10.1002/14651858.CD010296.pub2

For peer review only - http://bmjopen.bmj.com/site/about/guidelines.xhtml

17. Sommer WH, Hoffmann RT, Becker CR, et al. Comparison of time-resolved CT-angiography, contrast-enhanced ultrasound and digital subtraction angiography in a patient with a small type II endoleak after endovascular aneurysm repair. *Clinical hemorheology and microcirculation* 2010;45(1):19-25. doi: 10.3233/CH-2010-1283
18. Koike Y, Ishida K, Hase S, et al. Dynamic Volumetric CT Angiography for the Detection and Classification of Endoleaks: Application of Cine Imaging Using a 320-Row CT Scanner with 16-cm Detectors. *Journal of Vascular and Interventional Radiology* 2014;25(8):1172-80.e1. doi: http://dx.doi.org/10.1016/j.jvir.2014.03.019
19. Sommer WH, Becker CR, Haack M, et al. Time-resolved CT Angiography for the Detection and Classification of Endoleaks. *Radiology* 2012;263(3):917-26. doi: doi:10.1148/radiol.12111217
20. Lehmkuhl L, Andres C, Lücke C, et al. Dynamic CT Angiography after Abdominal Aortic Endovascular Aneurysm Repair: Influence of Enhancement Patterns and Optimal Bolus Timing on Endoleak Detection. *Radiology* 2013;268(3):890-99. doi: doi:10.1148/radiol.13120197
21. Bossuyt PM, Reitsma JB, Bruns DE, et al. STARD 2015: an updated list of essential items for reporting diagnostic accuracy studies. *BMJ* 2015;351:h5527. doi: 10.1136/bmj.h5527
22. Cohen JF, Korevaar DA, Altman DG, et al. STARD 2015 guidelines for reporting diagnostic accuracy studies: explanation and elaboration. *BMJ Open* 2016;6(11):e012799. doi: 10.1136/bmjopen-2016-012799
23. Harrison GJ, Oshin OA, Vallabhaneni SR, et al. Surveillance after EVAR based on duplex ultrasound and abdominal radiography. *European journal of vascular and endovascular surgery : the official journal of the European Society for Vascular Surgery* 2011;42(2):187-92. doi: 10.1016/j.ejvs.2011.03.027 [published Online First: 2011/05/07]
24. Sim J, Wright CC. The kappa statistic in reliability studies: use, interpretation, and sample size requirements. *Phys Ther* 2005;85(3):257-68.
25. Malhotra RK, Indrayan A. A simple nomogram for sample size for estimating sensitivity and specificity of medical tests. *Indian J Ophthalmol* 2010;58(6):519-22. doi: 10.4103/0301-4738.71699

For peer review only

Tables

TABLE 1: Inclusion Criteria

**Aged 18 or over**
**Able to give informed consent**
**Undergone an EVAR of infra-renal abdominal aortic aneurysm**
**Planned for CTA of EVAR**

For peer review only

TABLE 2: Exclusion Criteria

**Unable to receive CTA Contrast**<br>**Allergy,**<br>**Insufficient renal function for standard outpatient contrast study (eGFR <45)**<br>**Overactive thyroid gland**
**Unable to receive CEUS contrast**<br>**Previous reaction to Sonovue (Ultrasound Contrast)**<br>**Allergy to sulphur hexafluoride (used in electrical industry in circuit breakers, switch gears & electrical equipment)**<br>**Recent acute coronary syndrome or unstable angina, typical angina at rest or frequent or repeated angina/chest pain – all within previous 7 days**<br>**Recent coronary intervention**
**Previous embolization of artery in region of EVAR (affects imaging quality)**
**BMI >30 (affects imaging quality)**

For peer review only

Table 3: Settings and radiation exposure from arterial and time-resolved phases of CT angiography

Arterial Phase (outside Study)	Time-resolved phases (inside study)
120kV	Tube Voltage 80kV
230mAs (effective current – scanner automatically varies)	Tube Current 120mAs
variable (Dependant on body length)	Scan Length 27cm
One	Seven
599.6*	Expected DLP 78.9mGy/cm per phase (552.3mGy/cm for time-resolved phase)

*average DLP used for an arterial phase scan in all CTA scans in Royal Liverpool Hospital in month of July 2015.

For peer review only

Data Collection Pro-forma

Participant Study ID:

Participant Questionnaire:

Reason for further investigation:

Height (cm):
Weight (kg):
Blood Pressure:
Pulse rate:

Functional Status:
0 - you are fully active, more or less as you were before your illness

1 - you cannot carry out heavy physical work, but can do anything else

2 - you are up and about more than half the day. You can look after yourself, but cannot work

Estimated walking distance:

Previous Heart Disease:
Known to have AF or other arythmia: Yes/No
Known IHD (Angina, ACS, MI): Yes/No Details:…………………………………………….
Previous Treatments:

Patients had echo in past: Yes/No Details……………………………………..

Current Medications:

For peer review only

For peer review only - http://bmjopen.bmj.com/site/about/guidelines.xhtml

CDU:

Participant Study ID:

Completed by (investigators name):

Time start: Time Completed:

<u>Diagnostic images obtained of:</u>

Aortic neck	Yes/No		
Aneurysm body with graft in situ	Yes/No		
Bifurcation	Yes/No		
Right CIA (Midpoint of limb)	Yes/No	Left CIA (Midpoint of limb)	Yes/No
Right Limb/native transition	Yes/No	Left Limb/native transition	Yes/No

<u>Aortic Measurements:</u>

PSV in native aorta:

Native Aorta PI:

Neck / D2 diameter (mm):

Aneurysm /D3 diameter (mm):

Endoleak seen: Yes/No

Details:	<u>Endoleak 1</u>	<u>Endoleak 2</u>	<u>Endoleak 3</u>
Type:	Ia/Ib/II/III/Other	Ia/Ib/II/III/Other	Ia/Ib/II/III/Other
Inflow point(s):			
Outflow point(s):			
Certain of flow direction:	Yes/No	Yes/No	Yes/No

Limbs:

	<u>Right</u>	<u>Left</u>
Distal PSV measurement		
PI		
Wave form	Mono / Bi / Triphasic	Mono / Bi / Triphasic

Comments:

Common Femoral Arteries:

	<u>Right</u>	<u>Left</u>
Distal PSV measurement		
PI		
Wave form	Mono / Bi / Triphasic	Mono / Bi / Triphasic

Comments:

For peer review only

For peer review only - http://bmjopen.bmj.com/site/about/guidelines.xhtml

CEUS:

Participant Study ID:
Completed by (investigators name):
Time start: Time Completed:

First Contrast Injection: (all timings from start of contrast injection)

Time till seen in graft(s):
Time till seen in endoleak (s):

Contrast seen in Endoleak 1:	Yes / No	Endoleak 1 Type: Ia/Ib/II/III/Other
Contrast Seen in Endoleak 2:	Yes / No	Endoleak 2 Type: Ia/Ib/II/III/Other
Contrast seen in Endoleak 3:	Yes / No	Endoleak 3 Type: Ia/Ib/II/III/Other

Second Contrast Injection:

Time till seen in graft(s):
Time till seen in endoleak (s):

Contrast seen in Endoleak 1:	Yes / No	Endoleak 1 Type: Ia/Ib/II/III/Other
Contrast Seen in Endoleak 2:	Yes / No	Endoleak 2 Type: Ia/Ib/II/III/Other
Contrast seen in Endoleak 3:	Yes / No	Endoleak 3 Type: Ia/Ib/II/III/Other

For peer review only

For peer review only - http://bmjopen.bmj.com/site/about/guidelines.xhtml

Temporal CTA (in scan measurements)

Participant Study ID: Completed by (investigators name):

Time start: Time Completed:

Temporal CTA (reporting)

Completed by (investigators name):

Time reporting started: Time reporting finished:

Phase 2.5 Seconds

Image quality: Fully diagnostic 5 4 3 2 1 Non-diagnostic

Endoleak seen: Yes/No

Details:	Endoleak 1	Endoleak 2	Endoleak 3
Type:	Ia/Ib/II/III/Other	Ia/Ib/II/III/Other	Ia/Ib/II/III/Other
Inflow point(s):			
HU at inflow:			
Outflow point(s):			
HU at outflow:			
Certain of flow direction:	Yes/No	Yes/No	Yes/No

Hounsfield Unit measurements: (excluding any calcification of graft structures)

Inferior vena cava: .... HU

Aortic lumen at superior fabric markers of endograft: .... HU

Aortic lumen at bifurcation of endograft: .... HU

Iliac lumen at Distal end of right limb of endograft .... HU

Iliac lumen at distal end of left limb of endograft .... HU

Phase 5 Seconds

Image quality: Fully diagnostic 5 4 3 2 1 Non-diagnostic

Endoleak seen: Yes/No

Details:	Endoleak 1	Endoleak 2	Endoleak 3
Type:	Ia/Ib/II/III/Other	Ia/Ib/II/III/Other	Ia/Ib/II/III/Other
Inflow point(s):			
HU at inflow:			
Outflow point(s):			
HU at outflow:			
Certain of flow direction:	Yes/No	Yes/No	Yes/No

Hounsfield Unit measurements: (excluding any calcification of graft structures)

Inferior vena cava: .... HU

Aortic lumen at superior fabric markers of endograft: .... HU

Aortic lumen at bifurcation of endograft: .... HU

Iliac lumen at Distal end of right limb of endograft .... HU

Iliac lumen at distal end of left limb of endograft .... HU

For peer review only

For peer review only - http://bmjopen.bmj.com/site/about/guidelines.xhtml

Phase 7.5 Seconds

Image quality: Fully diagnostic 5 4 3 2 1 Non-diagnostic

Endoleak seen: Yes/No

Details:	Endoleak 1	Endoleak 2	Endoleak 3
Type:	Ia/Ib/II/III/Other	Ia/Ib/II/III/Other	Ia/Ib/II/III/Other
Inflow point(s):			
HU at inflow:			
Outflow point(s):			
HU at outflow:			
Certain of flow direction:	Yes/No	Yes/No	Yes/No

Hounsfield Unit measurements: (excluding any calcification of graft structures)

Inferior vena cava: .... HU

Aortic lumen at superior fabric markers of endograft: .... HU

Aortic lumen at bifurcation of endograft: .... HU

Iliac lumen at Distal end of right limb of endograft .... HU

Iliac lumen at distal end of left limb of endograft .... HU

Phase 10 Seconds

Image quality: Fully diagnostic 5 4 3 2 1 Non-diagnostic

Endoleak seen: Yes/No

Details:	Endoleak 1	Endoleak 2	Endoleak 3
Type:	Ia/Ib/II/III/Other	Ia/Ib/II/III/Other	Ia/Ib/II/III/Other
Inflow point(s):			
HU at inflow:			
Outflow point(s):			
HU at outflow:			
Certain of flow direction:	Yes/No	Yes/No	Yes/No

Hounsfield Unit measurements: (excluding any calcification of graft structures)

Inferior vena cava: .... HU

Aortic lumen at superior fabric markers of endograft: .... HU

Aortic lumen at bifurcation of endograft: .... HU

Iliac lumen at Distal end of right limb of endograft .... HU

Iliac lumen at distal end of left limb of endograft .... HU

For peer review only - http://bmjopen.bmj.com/site/about/guidelines.xhtml

Phase 15 Seconds

Image quality:	Fully diagnostic	5	4	3	2	1	Non-diagnostic

Endoleak seen: Yes/No

Details:	Endoleak 1	Endoleak 2	Endoleak 3
Type:	Ia/Ib/II/III/Other	Ia/Ib/II/III/Other	Ia/Ib/II/III/Other
Inflow point(s):			
HU at inflow:			
Outflow point(s):			
HU at outflow:			
Certain of flow direction:	Yes/No	Yes/No	Yes/No

Hounsfield Unit measurements: (excluding any calcification of graft structures)

Inferior vena cava: .... HU
Aortic lumen at superior fabric markers of endograft: .... HU
Aortic lumen at bifurcation of endograft: .... HU
Iliac lumen at Distal end of right limb of endograft .... HU
Iliac lumen at distal end of left limb of endograft .... HU

Phase 20 Seconds

Image quality:	Fully diagnostic	5	4	3	2	1	Non-diagnostic

Endoleak seen: Yes/No

Details:	Endoleak 1	Endoleak 2	Endoleak 3
Type:	Ia/Ib/II/III/Other	Ia/Ib/II/III/Other	Ia/Ib/II/III/Other
Inflow point(s):			
HU at inflow:			
Outflow point(s):			
HU at outflow:			
Certain of flow direction:	Yes/No	Yes/No	Yes/No

Hounsfield Unit measurements: (excluding any calcification of graft structures)

Inferior vena cava: .... HU
Aortic lumen at superior fabric markers of endograft: .... HU
Aortic lumen at bifurcation of endograft: .... HU
Iliac lumen at Distal end of right limb of endograft .... HU
Iliac lumen at distal end of left limb of endograft .... HU

For peer review only - http://bmjopen.bmj.com/site/about/guidelines.xhtml

Phase 25 Seconds

Image quality: Fully diagnostic 5 4 3 2 1 Non-diagnostic

Endoleak seen: Yes/No

Details:	Endoleak 1	Endoleak 2	Endoleak 3
Type:	Ia/Ib/II/III/Other	Ia/Ib/II/III/Other	Ia/Ib/II/III/Other
Inflow point(s):			
HU at inflow:			
Outflow point(s):			
HU at outflow:			
Certain of flow direction:	Yes/No	Yes/No	Yes/No

Hounsfield Unit measurements: (excluding any calcification of graft structures)

Inferior vena cava: .... HU
Aortic lumen at superior fabric markers of endograft: .... HU
Aortic lumen at bifurcation of endograft: .... HU
Iliac lumen at Distal end of right limb of endograft .... HU
Iliac lumen at distal end of left limb of endograft .... HU

Venous Phase

Image quality: Fully diagnostic 5 4 3 2 1 Non-diagnostic

Endoleak seen: Yes/No

Details:	Endoleak 1	Endoleak 2	Endoleak 3
Type:	Ia/Ib/II/III/Other	Ia/Ib/II/III/Other	Ia/Ib/II/III/Other
Inflow point(s):			
HU at inflow:			
Outflow point(s):			
HU at outflow:			
Certain of flow direction:	Yes/No	Yes/No	Yes/No

Hounsfield Unit measurements: (excluding any calcification of graft structures)

Inferior vena cava: .... HU
Aortic lumen at superior fabric markers of endograft: .... HU
Aortic lumen at bifurcation of endograft: .... HU
Iliac lumen at Distal end of right limb of endograft .... HU
Iliac lumen at distal end of left limb of endograft .... HU

Other comments / diagnostic findings:

For peer review only

For peer review only - http://bmjopen.bmj.com/site/about/guidelines.xhtml

Included

Section & Topic	No	Item	
**TITLE OR ABSTRACT**			
	1	Identification as a study of diagnostic accuracy using at least one measure of accuracy (such as sensitivity, specificity, predictive values, or AUC)	✓
**ABSTRACT**			
	2	Structured summary of study design, methods, results, and conclusions (for specific guidance, see STARD for Abstracts)	NA Protocol paper
**INTRODUCTION**			
	3	Scientific and clinical background, including the intended use and clinical role of the index test	✓
	4	Study objectives and hypotheses	✓
**METHODS**			
Study design	5	Whether data collection was planned before the index test and reference standard were performed (prospective study) or after (retrospective study)	✓
Participants	6	Eligibility criteria	✓
	7	On what basis potentially eligible participants were identified (such as symptoms, results from previous tests, inclusion in registry)	✓
	8	Where and when potentially eligible participants were identified (setting, location and dates)	✓
	9	Whether participants formed a consecutive, random or convenience series	✓
Test methods	10a	Index test, in sufficient detail to allow replication	✓
	10b	Reference standard, in sufficient detail to allow replication	✓
	11	Rationale for choosing the reference standard (if alternatives exist)	✓
	12a	Definition of and rationale for test positivity cut-offs or result categories of the index test, distinguishing pre-specified from exploratory	✓
	12b	Definition of and rationale for test positivity cut-offs or result categories of the reference standard, distinguishing pre-specified from exploratory	✓
	13a	Whether clinical information and reference standard results were available to the performers/readers of the index test	✓
	13b	Whether clinical information and index test results were available to the assessors of the reference standard	✓
Analysis	14	Methods for estimating or comparing measures of diagnostic accuracy	✓
	15	How indeterminate index test or reference standard results were handled	✓
	16	How missing data on the index test and reference standard were handled	NA.
	17	Any analyses of variability in diagnostic accuracy, distinguishing pre-specified from exploratory	NA.
	18	Intended sample size and how it was determined	✓
**RESULTS**			
Participants	19	Flow of participants, using a diagram	NA (items 19–25)
	20	Baseline demographic and clinical characteristics of participants	
	21a	Distribution of severity of disease in those with the target condition	
	21b	Distribution of alternative diagnoses in those without the target condition	
	22	Time interval and any clinical interventions between index test and reference standard	
Test results	23	Cross tabulation of the index test results (or their distribution) by the results of the reference standard	
	24	Estimates of diagnostic accuracy and their precision (such as 95% confidence intervals)	
	25	Any adverse events from performing the index test or the reference standard	
**DISCUSSION**			
	26	Study limitations, including sources of potential bias, statistical uncertainty, and generalisability	
	27	Implications for practice, including the intended use and clinical role of the index test	NA
**OTHER INFORMATION**			
	28	Registration number and name of registry	✓
	29	Where the full study protocol can be accessed	NA.
	30	Sources of funding and other support; role of funders	✓

cc BY SA

BMJ Open

Protocol: A Prospective, Single UK Centre, Comparative study of the Predictive Values of Contrast Enhanced Ultrasound compared to Time-Resolved Computer Tomography Angiography in the Detection and Characterisation of Endoleaks in High Risk Endovascular Aneurysm Repair Surveillance Patients

Journal:	*BMJ Open*
Manuscript ID	bmjopen-2017-020835.R1
Article Type:	Protocol
Date Submitted by the Author:	31-Jan-2018

Complete List of Authors:	Roy, Iain; University of Liverpool Institute of Ageing and Chronic Disease; Royal Liverpool University Hospital, Liverpool Vascular & Endovascular Service<br>Chan , Tze; Royal Liverpool University Hospital, Department of Radiology<br>Czanner, Gabriela; University of Liverpool Institute of Ageing and Chronic Disease; University of Liverpool, Department of Biostatistics<br>Wallace, Stven; Royal Liverpool University Hospital, Liverpool Vascular & Endovascular Service<br>Vallabhaneni, Srinivasa; University of Liverpool Institute of Ageing and Chronic Disease; Royal Liverpool University Hospital, Liverpool Vascular & Endovascular Service

**Primary Subject Heading**:	Surgery
Secondary Subject Heading:	Cardiovascular medicine, Radiology and imaging
Keywords:	Ultrasound < RADIOLOGY & IMAGING, VASCULAR SURGERY, Cardiovascular imaging < RADIOLOGY & IMAGING, Computed tomography < RADIOLOGY & IMAGING, Aneurysm, Diagnostic radiology < RADIOLOGY & IMAGING

SCHOLARONE™
Manuscripts

Full Title:

Protocol: A Prospective, Single UK Centre, Comparative study of the Predictive Values of Contrast Enhanced Ultrasound compared to Time-Resolved Computer Tomography Angiography in the Detection and Characterisation of Endoleaks in High Risk Endovascular Aneurysm Repair Surveillance Patients

Short Title: Contrast Enhanced Ultrasound, True Endoleak Detection rate

Authors:

Iain N Roy MBChB MRCS[1,2], Tze Yuan Chan MBChB FRCR[3], Gabriela Czanner PhD[1,4], Steve Wallace BSc AVS[2], Srinivasa Rao Vallabhaneni MD, FRCS[1,2].

1. Institute of Ageing & Chronic Disease, University of Liverpool, UK
2. Liverpool Vascular & Endovascular Service, Royal Liverpool University Hospital, UK
3. Department of Radiology, Royal Liverpool University Hospital, UK
4. Department of Biostatistics, University of Liverpool, UK

Correspondence:

Mr Iain Roy

Liverpool Vascular & Endovascular Service (LiVES)

Royal Liverpool Hospital

Prescot Street

Liverpool

L7 8XP

Telephone: +44 151 706 3457

Fax : +44 151 706 5827

E-mail: iain.roy@liverpool.ac.uk

Article Word Count: 3002 words

For peer review only

Journal Subject Codes: Diagnostic Testing, Imaging, Ultrasound, Aneurysm

Abstract

Introduction

Diagnosis of endoleaks is imperative to prevent failure of endovascular aneurysm repairs (EVAR). The gold standard for diagnosis of endoleaks is catheter directed subtraction angiography, which is not a practicable choice for surveillance. Computer Tomography Angiography (CTA) is the historical surveillance modality of choice. Concerns over cost, potential nephrotoxicity of contrast agents and repeated radiation exposure led to Colour Duplex Ultrasound Scan (CDUS) becoming an established alternative. CDUS has a lower sensitivity and specificity for endoleaks detection compared to CTA. Contrast Enhanced Ultrasound Scan (CEUS) represents an improvement of ultrasound imaging but comparisons against CTA report widely varying results, likely due to technical factors of CEUS and limitations of single phase CTA.

The development of time-resolved CTA (tCTA) offers timing information that much more closely mirrors the dynamic information available from CEUS. Theoretically these two imaging modalities have the best potential for diagnostic accuracy. The aim of this study will be to compare CEUS to tCTA and investigate the utility of other measurements available from tCTA.

Methods and Analysis

This is a prospective, single UK centre, comparative study of paired binary diagnostic imaging modalities. Patients identified in routine post EVAR surveillance as at risk of having a graft-related endoleak will undergo a CEUS and time-resolved CTA (tCTA) on the same day. This will allow the first comparison of CEUS to a semi-dynamic form of CTA. CEUS sensitivity and specificity to endoleak detection will be calculated.

Ethics and dissemination

For peer review only

The study has achieved ethical approval. We hope the results will define the diagnostic accuracy of CEUS in comparison to a semi-dynamic form of CTA, representing a methodological improvement from previous studies. Results will be submitted for presentation at national and international Vascular Surgery & Radiology meetings. The full results are planned to be published in a medical journal.

Trial registration

ClinicalTrials.gov: NCT02688751

For peer review only

Strengths and limitations of this study

Strengths

- First comparison of Contrast Enhanced Ultrasound to a dynamic form of CT imaging, representing a methodological improvement
- Both imaging modalities occur on the same day removing changing findings as a confounding factor.
- Primary outcome set as Type I/III endoleak (most clinically significant) rather than all endoleaks

Limitations

- Appropriately powered but small study
- Single Centre study

For peer review only

Introduction

Background and rationale

Endovascular aneurysm repair (EVAR) is the intervention of choice to treat abdominal aortic aneurysms (AAA).[1] In comparison to open surgical repair (OSR), EVAR confers a reduction of mortality lasting into the short to intermediate term.[2] However, EVAR is associated with complications which sometimes require secondary interventions in order to maintain efficacy of EVAR. This has been recognised since the inception of the technique and confirmed in observational studies as well as RCT s.[3-6] Therefore periodic surveillance imaging is recommended for life following EVAR.[7 8] The importance of post-EVAR surveillance remains enduring, its value further highlighted by a recently published analysis of 15-year follow-up after EVAR[2] .

The commonest complication in EVAR surveillance is an endoleak,[9] which is "persistent blood flow within the aneurysm sac but outside the stent-graft".[10] Endoleaks are classified based on the source of blood flow ,[10] but can be grouped into stent-graft related (Types I and III) and type II (non stent-graft related) endoleaks. Stent-graft related endoleaks generally transmit high pressure causing a high risk of aneurysm expansion/rupture (treatment failure)[11 12]. In contrast, type II endoleaks generally run a benign course, particularly in the absence of aneurysm expansion.[12] With regards to endoleak imaging, high sensitivity of detection and high specificity of characterisation improve diagnostic utility of surveillance, in particular with an emphasis on distinguishing stent-graft related endoleaks from type II endoleaks. Digital subtraction angiography in multiple planes and a high frame rate of acquisition is the gold standard of endoleak imaging; high frame rates to demonstrate endoleak heamodynamics for better characterisation. However, this modality is not tenable to be used as surveillance imaging.

Historically EVAR surveillance was undertaken using Computer Tomography Angiography (CTA). Arterial phase CT was the most frequently used modality, although selectively or routinely additional phases are used such as un-enhanced, venous phase and even delayed phase. Concerns over cost,

For peer review only

use of potentially nephrotoxic contrast agent and repeated radiation exposure led to alternative imaging modalities being investigated and implemented in surveillance regimens. Colour Duplex Ultrasound Scan (CDUS) is the most widely used imaging modality currently.[13] CDUS is reported to have a lower sensitivity and specificity to detect stent-graft related endoleaks compared to CTA. [14]

Contrast Enhanced Ultrasound Scan (CEUS) has been investigated as an adjunct to CDUS in the hope of improving sensitivity to endoleak detection. CEUS involves intravenous injection of a microbubble contrast which remains in the blood, allowing improved detection of endoleaks, particularly with contrast coherent ultrasound imaging. CEUS also allows continuous (dynamic) or real time monitoring of the aneurysm and endoleak as the contrast agent arrives into the endoleak. Modern microbubble agents are expired by the respiratory system, thus avoiding nephrotoxicity. A recent review of 30,222 administrations of a CEUS contrast agent demonstrated a low adverse reaction rate of 0.020%.[15] CEUS also obviates the radiation exposure associated with CTA. 3D acquisition and reconstructions of CEUS scans are possible,[16] however this development in the technology is currently limited to single phases of acquisition and therefore losses the dynamic information available in standard CEUS.

Systematic review of the diagnostic accuracy of CEUS (in comparison to CTA) in detection of any endoleak revealed variable sensitivity ranging from 67- 100% and specificity ranging from 79-100%.[17] The value of this review is compromised by heterogeneity of contrast material used and non-reporting of ultrasound imaging technique used, specifically whether colour duplex combined with contrast or harmonic CEUS imaging was used. There are potentially additional reasons variability in the reporting such as operator dependence, quality of equipment used as well as body habitus of the patient. The consideration that neither CEUS nor single phase CTA represent gold standard of endoleak diagnosis led to the metanalysis[14 17] adopting a bivariate model of analysis. This approach does not compensate for the lack of gold standard comparator as it favours any modality producing

For peer review only

false positives. Comparison against the gold standard has never been established for either modality.

Time-resolved CT

Time-resolved CTA (tCTA) was first described for endoleak detection in 2010[18]. The single arterial phase is replaced by multiple phases in tCTA, which are typically of lower radiation dose, thus offering dynamic observations of endoleaks, such as flow direction and filling speed, while still retaining many of the advantages of CTA (3D reconstruction etc.) which closely mirror the advantages of the Multi-planar Digital subtraction catheter angiography. The multiple phases of tCTA can be achieved by broad CT detectors and a static patient in the Fowler position[19] or rapid shuttling of a supine patient through a standard detector[20 21]. Sufficient amount of measurements regarding filling patterns of endoleaks on tCTA[21] are now available to be able to replace a standard arterial phase in CTA with a tCTA that is aimed at detecting stent-graft related endoleaks, without increasing radiation exposure for the patient. Now timings and interpretation of tCTA are understood it is timely to do a comparison of CEUS to the improved comparator of tCTA. This overcomes the limitations of previous studies by comparison of CEUS to a (semi) dynamic form of CTA imaging as a gold standard.

Methods

Study Design

This is a prospective single centre comparative study of paired diagnostic imaging modalities, designed to comply with the “Standards for Reporting Diagnostic accuracy studies”.[22 23] Participants will be recruited from a city-wide vascular service in the UK. The service is arranged in a hub and spoke configuration locally and regularly accepts tertiary referrals for complications of previous aortic surgery at other centres. EVAR surveillance is predominately undertaken using CDUS and plain radiography.[24]

For peer review only

Participants

Potential participants are referred to the study by their vascular surgeon when they require a CTA for further investigations of an endoleak following routine EVAR surveillance and meet inclusion criteria (TABLE 1). They are typically patients with a suspicion of a graft related endoleak or aneurysm expansion on CDUS surveillance. They are then assessed by the study for eligibility based on exclusion criteria (TABLE 2) and approached to participate in the study, if appropriate. Patients are approached by an investigator and those who give their written informed consent to participate will be enrolled in the study.

Test Methods

Participants attend and have a CEUS (index test) and tCTA (reference standard) on the same day. This represents a change from standard care for participants who would otherwise have a CEUS and triple phase CTA (non-contrast, arterial (20s) and delayed venous phase (90s)) often on separate days. Participants will also be asked a short number of closed questions to assess functional status and cardiac function (supplementary material). Upon completion of the CEUS & tCTA, participants are returned to their referring surgeons, with clinical reports of the studies for ongoing care. The study team follows the participants further treatment and investigation until the end of the study.

Contrast Enhanced Ultrasound (CEUS)

Contrast enhanced ultrasound is performed in combination with a standard Colour Duplex Ultrasound Scan in our institution. It is reported as a binary test yielding two values: present or absent for each endoleak type. It is conducted by an experienced Clinical Vascular Scientist with extensive involvement in scans for EVAR surveillance. It is performed on a Philips IU22 ultrasound machine (Philips, Amsterdam, Netherlands), using the a 2-5MHz abdominal curved array probe. Grey scale images of the aneurysm neck (when possible), iliac seal zones and maximum aneurysm dimensions are obtained and measured in maximum antero-posterior and medio-lateral dimensions. Note is made of the echogenicity of thrombus within the aneurysm sac. Using colour flow imaging

For peer review only

and spectral Doppler, waveform characteristics and velocities are recorded in the common femoral arteries. The stent-graft is interrogated using colour and spectral Doppler to ascertain patency and flow heamodynamics of the neck, main body as well as both limbs. Any abnormalities in these parameters are reported. Colour Doppler is used to detect any endoleak. If present, its type, point of inflow, point of outflow and flow dynamics (using sectoral doppler) are reported.

Optimum views of the area of concern are obtained, prior to contrast injection, using appropriate machine set ups and controls as determined by the operator. 2.4mls Sulphur Hexafluoride Microbubble Contrast (SonoVue™, Bracco, Milan, Italy) is injected followed by 10mls of sodium chloride 0.9%, the on-screen timer is started at the start of the injection. Flow direction and filling time ideally should be determined and anatomy of the endoleak established by interrogation. Passive elimination of the contrast agent is allowed to occur and the process repeated for a second injection.

CEUS scan will be reported by the performing vascular scientist to the data point recorded in the data collection proforma (supplementary material), in addition to any clinically relevant points. The vascular scientist will be blinded to the concurrent tCTA at the time of reporting, although will be aware of the previous findings on EVAR surveillance.

Time-resolved Computer Tomography Angiography (tCTA)

tCTA is performed on a Siemens Definition AS+ scanner (Seimens, Munich, Germany), in our institution. Participants are positioned supine with arms raised above their head. The contrast injector is connected to a 20G (or larger) IV catheter in an anterior cubital fossa vein. A standard topogram scan is performed. Unless not required, a non-contrast scan is performed. The maximum length that can be covered for the time resolution required is 27cm. This is centred over the EVAR stent-graft. Abdominal guides are placed at upper aspect of diaphragm and common femoral arteries for venous phase of scan. Abdominal aorta, just proximal to EVAR graft is selected as trigger area for time-resolved phases.

For peer review only

Contrast is injected, using an auto injector, at 4ml/s for 96mls. Participants are asked to adopt shallow breaths and not hold their breath. The time-resolved phase is triggered by a HU>90 in trigger area. Phases occur at 2.5, 5, 7.5, 10, 15, 20 and 25 seconds following the automatic trigger, these occur in cranio-caudually acquisition, except the 5 & 7.5 second phases which occur caudo-cranially direction. The venous phase is taken in full inspiration and is acquired 75s following the trigger. Tube setting and calculated predicted radiation exposure are presented in Table 3.

All tCTA scans will be reported by a single consultant vascular radiologist. This reporter will be blinded to the results of the CEUS and collect data to the proforma. It will be reported as a binary test yielding two values: present or absent for each type of endoleak.

Outcome Measures

The primary outcome is:

The predictive values of CEUS in comparison to tCTA (as comparator) to detect stent-graft related endoleaks.

Secondary outcomes are:

1. Any adverse events during CEUS or tCTA.
2. Predictive values of CEUS in comparison to tCTA to detect type II endoleaks
3. Predictive values of both tCTA & CEUS in predicting final endoleak diagnosis (following any further investigations).
4. Predictive values of both tCTA & CEUS in predicting need for a secondary intervention.
5. Evaluate the association between CEUS temporal delay (difference between contrast in endograft & contrast in endoleak) and evaluate its ability to improve the differentiation of endoleak type.
6. Evaluate the association between "CEUS contrast in endoleak" to "tCTA contrast in endoleak" and assess potential as predictive tool, for optimum timing of CTA phases.

For peer review only

Analysis Plan

Associations will be established / refuted with summary statistics and graphical analysis and appropriate further statistical testing within the framework of logistic regression. If association can be established then predictive modelling will be undertaken. The agreement between CEUS and tCTA will be evaluated with Kappa statistic.[25] Sensitivity/specificity will be calculated along with binomial exact 95% confidence intervals and leave-one-out cross-validation. We will also report the positive predictive value and negative predictive value.

The power calculation,[26] showed the required sample size to be 74. This was calculated based on a prevalence of stent-graft related endoleaks of 11% as demonstrated on previous tCTA studies of endoleaks. It was powered to detect a predicted sensitivity of 0.95 with a tolerated confidence interval of ±0.15. The study commenced recruitment in February 2016 and is on-going.

Monitoring & Data

All patients referred to the study are recorded in the screening log. The sponsoring trust will provide governance oversight and annual reports to the REC will provide ethical overview. Radiation exposure of participants will be monitored and reported to the sponsors and approving research ethics committee. No interim analysis is planned.

Upon completion of the study, all identifiable patient demographics will be removed and anonymised data will be stored to allow for future analysis of unforeseen benefits. During the study, data is stored in a secure manner within the hosts institutions data management processes. Access is restricted to the investigators and for audit by the sponsor.

Ethics and dissemination

The main ethical consideration is the change of care from CTA to a tCTA, this was felt to be appropriate in the context of informed consent, now it can be performed without increasing radiation exposure. Ethical approval (15/NW/0908) was granted by a NHS Research Ethics

For peer review only

Committee. Upon completion of analysis we expect to publish in a medical journal, along with the anonymised data set and present the findings widely.

For peer review only

Authors' contributions

IR – Developed and wrote the protocol

TC – Reviewed and revised the radiological/imaging elements of the protocol

SW – Reviewed and revised the ultrasound elements of the protocol

GC – Reviewed and revised the statistical analysis elements of the protocol

SV – Conceived the project and supervised IR in developing the protocol

Funding statement

This research received no specific grant from any funding agency in the public, commercial or not-for-profit sectors.

It is sponsored by: Royal Liverpool & Broadgreen University Hospitals Hospitals NHS Trust (Ref: 5083) and supported by use of staff and resources from The Royal Liverpool University Hospital, Liverpool, UK and The Institute of Ageing & Chronic Disease, The University of Liverpool, UK.

Competing interest's statement

None

For peer review only

References

1. Waton S JA, Heikkila K, Cromwell D, Loftus I. National Vascular Registry: 2016 Annual report. London: The Royal College of Surgeons of England, November 2016.
2. Patel R, Sweeting MJ, Powell JT, et al. Endovascular versus open repair of abdominal aortic aneurysm in 15-years' follow-up of the UK endovascular aneurysm repair trial 1 (EVAR trial 1): a randomised controlled trial. *The Lancet*;388(10058):2366-74. doi: 10.1016/S0140-6736(16)31135-7
3. Blankensteijn JD, de Jong SECA, Prinssen M, et al. Two-Year Outcomes after Conventional or Endovascular Repair of Abdominal Aortic Aneurysms. *New England Journal of Medicine* 2005;352(23):2398-405. doi: 10.1056/NEJMoa051255
4. Becquemin JP, Pillet Jc Fau - Lescalie F, Lescalie F Fau - Sapoval M, et al. A randomized controlled trial of endovascular aneurysm repair versus open surgery for abdominal aortic aneurysms in low- to moderate-risk patients. (1097-6809 (Electronic))
5. Lederle FA, Freischlag JA, Kyriakides TC, et al. Long-Term Comparison of Endovascular and Open Repair of Abdominal Aortic Aneurysm. *New England Journal of Medicine* 2012;367(21):1988-97. doi: 10.1056/NEJMoa1207481
6. Investigators TUKET. Endovascular versus Open Repair of Abdominal Aortic Aneurysm. *New England Journal of Medicine* 2010;362(20):1863-71. doi: 10.1056/NEJMoa0909305
7. Moll FL, Powell JT, Fraedrich G, et al. Management of abdominal aortic aneurysms clinical practice guidelines of the European society for vascular surgery. *Eur J Vasc Endovasc Surg* 2011;41 Suppl 1:S1-S58. doi: 10.1016/j.ejvs.2010.09.011
8. Chaikof EL, Brewster DC, Dalman RL, et al. SVS practice guidelines for the care of patients with an abdominal aortic aneurysm: executive summary. *J Vasc Surg* 2009;50(4):880-96. doi: 10.1016/j.jvs.2009.07.001
9. Hobo R, Buth J, collaborators E. Secondary interventions following endovascular abdominal aortic aneurysm repair using current endografts. A EUROSTAR report. *J Vasc Surg* 2006;43(5):896-902. doi: 10.1016/j.jvs.2006.01.010
10. White GH, May J, Waugh RC, et al. Type III and type IV endoleak: toward a complete definition of blood flow in the sac after endoluminal AAA repair. *J Endovasc Surg* 1998;5(4):305-9. doi: 10.1583/1074-6218(1998)005<0305:TIATIE>2.0.CO;2
11. Veith FJ, Baum RA, Ohki T, et al. Nature and significance of endoleaks and endotension: summary of opinions expressed at an international conference. *J Vasc Surg* 2002;35(5):1029-35.
12. Harris PL, Vallabhaneni SR, Desgranges P, et al. Incidence and risk factors of late rupture, conversion, and death after endovascular repair of infrarenal aortic aneurysms: The EUROSTAR experience. *Journal of Vascular Surgery*;32(4):739-49. doi: 10.1067/mva.2000.109990
13. Karthikesalingam A, Page AA, Pettengell C, et al. Heterogeneity in surveillance after endovascular aneurysm repair in the UK. *Eur J Vasc Endovasc Surg* 2011;42(5):585-90. doi: 10.1016/j.ejvs.2011.06.053
14. Karthikesalingam A, Al-Jundi W, Jackson D, et al. Systematic review and meta-analysis of duplex ultrasonography, contrast-enhanced ultrasonography or computed tomography for surveillance after endovascular aneurysm repair. *The British journal of surgery* 2012;99(11):1514-23. doi: 10.1002/bjs.8873 [published Online First: 2012/09/25]
15. Tang C, Fang K, Guo Y, et al. Safety of Sulfur Hexafluoride Microbubbles in Sonography of Abdominal and Superficial Organs: Retrospective Analysis of 30,222 Cases. *Journal of ultrasound in medicine : official journal of the American Institute of Ultrasound in Medicine* 2017;36(3):531-38. doi: 10.7863/ultra.15.11075
16. Lowe C, Abbas A, Rogers S, et al. Three-dimensional contrast-enhanced ultrasound improves endoleak detection and classification after endovascular aneurysm repair. *Journal of Vascular Surgery* 2017;65(5):1453-59. doi: https://doi.org/10.1016/j.jvs.2016.10.082

For peer review only

For peer review only - http://bmjopen.bmj.com/site/about/guidelines.xhtml

17. Abraha I, Luchetta ML, De Florio R, et al. Ultrasonography for endoleak detection after endoluminal abdominal aortic aneurysm repair. *Cochrane Database Syst Rev* 2017;6:CD010296. doi: 10.1002/14651858.CD010296.pub2
18. Sommer WH, Hoffmann RT, Becker CR, et al. Comparison of time-resolved CT-angiography, contrast-enhanced ultrasound and digital subtraction angiography in a patient with a small type II endoleak after endovascular aneurysm repair. *Clinical hemorheology and microcirculation* 2010;45(1):19-25. doi: 10.3233/CH-2010-1283
19. Koike Y, Ishida K, Hase S, et al. Dynamic Volumetric CT Angiography for the Detection and Classification of Endoleaks: Application of Cine Imaging Using a 320-Row CT Scanner with 16-cm Detectors. *Journal of Vascular and Interventional Radiology* 2014;25(8):1172-80.e1. doi: http://dx.doi.org/10.1016/j.jvir.2014.03.019
20. Sommer WH, Becker CR, Haack M, et al. Time-resolved CT Angiography for the Detection and Classification of Endoleaks. *Radiology* 2012;263(3):917-26. doi: doi:10.1148/radiol.12111217
21. Lehmkuhl L, Andres C, Lücke C, et al. Dynamic CT Angiography after Abdominal Aortic Endovascular Aneurysm Repair: Influence of Enhancement Patterns and Optimal Bolus Timing on Endoleak Detection. *Radiology* 2013;268(3):890-99. doi: doi:10.1148/radiol.13120197
22. Bossuyt PM, Reitsma JB, Bruns DE, et al. STARD 2015: an updated list of essential items for reporting diagnostic accuracy studies. *BMJ* 2015;351:h5527. doi: 10.1136/bmj.h5527
23. Cohen JF, Korevaar DA, Altman DG, et al. STARD 2015 guidelines for reporting diagnostic accuracy studies: explanation and elaboration. *BMJ Open* 2016;6(11):e012799. doi: 10.1136/bmjopen-2016-012799
24. Harrison GJ, Oshin OA, Vallabhaneni SR, et al. Surveillance after EVAR based on duplex ultrasound and abdominal radiography. *European journal of vascular and endovascular surgery : the official journal of the European Society for Vascular Surgery* 2011;42(2):187-92. doi: 10.1016/j.ejvs.2011.03.027 [published Online First: 2011/05/07]
25. Sim J, Wright CC. The kappa statistic in reliability studies: use, interpretation, and sample size requirements. *Phys Ther* 2005;85(3):257-68.
26. Malhotra RK, Indrayan A. A simple nomogram for sample size for estimating sensitivity and specificity of medical tests. *Indian J Ophthalmol* 2010;58(6):519-22. doi: 10.4103/0301-4738.71699

For peer review only

Tables

TABLE 1: Inclusion Criteria

**Aged 18 or over**
**Able to give informed consent**
**Undergone an EVAR of infra-renal abdominal aortic aneurysm**
**Planned for CTA of EVAR**

For peer review only

TABLE 2: Exclusion Criteria

**Unable to receive CTA Contrast**<br><br>**Allergy,**<br><br>**Insufficient renal function for standard outpatient contrast study (eGFR <45)**<br><br>**Overactive thyroid gland**
**Unable to receive CEUS contrast**<br><br>**Previous reaction to Sonovue (Ultrasound Contrast)**<br><br>**Allergy to sulphur hexafluoride (used in electrical industry in circuit breakers, switch gears & electrical equipment)**<br><br>**Recent acute coronary syndrome or unstable angina, typical angina at rest or frequent or repeated angina/chest pain – all within previous 7 days**<br><br>**Recent coronary intervention**
**Previous embolization of artery in region of EVAR (affects imaging quality)**
**BMI >30 (affects imaging quality)**

For peer review only

Table 3: Settings and radiation exposure from arterial and time-resolved phases of CT angiography

Arterial Phase (outside Study)	Time-resolved phases (inside study)
120kV	Tube Voltage 80kV
230mAs (effective current – scanner automatically varies)	Tube Current 120mAs
variable (Dependant on body length)	Scan Length 27cm
One	Seven
599.6*	Expected DLP 78.9mGy/cm per phase (552.3mGy/cm for time-resolved phase)

*average DLP used for an arterial phase scan in all CTA scans in Royal Liverpool Hospital in month of July 2015.

For peer review only

Data Collection Pro-forma

Participant Study ID:

Participant Questionnaire:

Reason for further investigation:

Height (cm):
Weight (kg):
Blood Pressure:
Pulse rate:

Functional Status:
0 - you are fully active, more or less as you were before your illness

1 - you cannot carry out heavy physical work, but can do anything else

2 - you are up and about more than half the day. You can look after yourself, but cannot work

Estimated walking distance:

Previous Heart Disease:
Known to have AF or other arythmia: Yes/No
Known IHD (Angina, ACS, MI): Yes/No Details:………………………………………….
Previous Treatments:

Patients had echo in past: Yes/No Details……………………………………..

Current Medications:

For peer review only

For peer review only - http://bmjopen.bmj.com/site/about/guidelines.xhtml

CDU:

Participant Study ID:
Completed by (investigators name):
Time start: Time Completed:

Diagnostic images obtained of:

Aortic neck	Yes/No		
Aneurysm body with graft in situ	Yes/No		
Bifurcation	Yes/No		
Right CIA (Midpoint of limb)	Yes/No	Left CIA (Midpoint of limb)	Yes/No
Right Limb/native transition	Yes/No	Left Limb/native transition	Yes/No

Aortic Measurements:
PSV in native aorta:
Native Aorta PI:
Neck / D2 diameter (mm):
Aneurysm /D3 diameter (mm):

Endoleak seen: Yes/No

Details:	Endoleak 1	Endoleak 2	Endoleak 3
Type:	Ia/Ib/II/III/Other	Ia/Ib/II/III/Other	Ia/Ib/II/III/Other
Inflow point(s):			
Outflow point(s):			
Certain of flow direction:	Yes/No	Yes/No	Yes/No

Limbs:

	Right	Left
Distal PSV measurement		
PI		
Wave form	Mono / Bi / Triphasic	Mono / Bi / Triphasic

Comments:

Common Femoral Arteries:

	Right	Left
Distal PSV measurement		
PI		
Wave form	Mono / Bi / Triphasic	Mono / Bi / Triphasic

Comments:

For peer review only - http://bmjopen.bmj.com/site/about/guidelines.xhtml

CEUS:

Participant Study ID:
Completed by (investigators name):
Time start: Time Completed:

First Contrast Injection: (all timings from start of contrast injection)

Time till seen in graft(s):
Time till seen in endoleak (s):

Contrast seen in Endoleak 1:	Yes / No	Endoleak 1 Type: Ia/Ib/II/III/Other
Contrast Seen in Endoleak 2:	Yes / No	Endoleak 2 Type: Ia/Ib/II/III/Other
Contrast seen in Endoleak 3:	Yes / No	Endoleak 3 Type: Ia/Ib/II/III/Other

Second Contrast Injection:

Time till seen in graft(s):
Time till seen in endoleak (s):

Contrast seen in Endoleak 1:	Yes / No	Endoleak 1 Type: Ia/Ib/II/III/Other
Contrast Seen in Endoleak 2:	Yes / No	Endoleak 2 Type: Ia/Ib/II/III/Other
Contrast seen in Endoleak 3:	Yes / No	Endoleak 3 Type: Ia/Ib/II/III/Other

For peer review only

For peer review only - http://bmjopen.bmj.com/site/about/guidelines.xhtml

Temporal CTA (in scan measurements)

Participant Study ID: Completed by (investigators name):

Time start: Time Completed:

Temporal CTA (reporting)

Completed by (investigators name):

Time reporting started: Time reporting finished:

Phase 2.5 Seconds

Image quality: Fully diagnostic 5 4 3 2 1 Non-diagnostic

Endoleak seen: Yes/No

Details:	Endoleak 1	Endoleak 2	Endoleak 3
Type:	Ia/Ib/II/III/Other	Ia/Ib/II/III/Other	Ia/Ib/II/III/Other
Inflow point(s):			
HU at inflow:			
Outflow point(s):			
HU at outflow:			
Certain of flow direction:	Yes/No	Yes/No	Yes/No

Hounsfield Unit measurements: (excluding any calcification of graft structures)

Inferior vena cava: .... HU

Aortic lumen at superior fabric markers of endograft: .... HU

Aortic lumen at bifurcation of endograft: .... HU

Iliac lumen at Distal end of right limb of endograft .... HU

Iliac lumen at distal end of left limb of endograft .... HU

Phase 5 Seconds

Image quality: Fully diagnostic 5 4 3 2 1 Non-diagnostic

Endoleak seen: Yes/No

Details:	Endoleak 1	Endoleak 2	Endoleak 3
Type:	Ia/Ib/II/III/Other	Ia/Ib/II/III/Other	Ia/Ib/II/III/Other
Inflow point(s):			
HU at inflow:			
Outflow point(s):			
HU at outflow:			
Certain of flow direction:	Yes/No	Yes/No	Yes/No

Hounsfield Unit measurements: (excluding any calcification of graft structures)

Inferior vena cava: .... HU

Aortic lumen at superior fabric markers of endograft: .... HU

Aortic lumen at bifurcation of endograft: .... HU

Iliac lumen at Distal end of right limb of endograft .... HU

Iliac lumen at distal end of left limb of endograft .... HU

For peer review only

For peer review only - http://bmjopen.bmj.com/site/about/guidelines.xhtml

Phase 7.5 Seconds

Image quality: Fully diagnostic 5 4 3 2 1 Non-diagnostic

Endoleak seen: Yes/No

Details:	Endoleak 1	Endoleak 2	Endoleak 3
Type:	Ia/Ib/II/III/Other	Ia/Ib/II/III/Other	Ia/Ib/II/III/Other
Inflow point(s):			
HU at inflow:			
Outflow point(s):			
HU at outflow:			
Certain of flow direction:	Yes/No	Yes/No	Yes/No

Hounsfield Unit measurements: (excluding any calcification of graft structures)

Inferior vena cava: .... HU
Aortic lumen at superior fabric markers of endograft: .... HU
Aortic lumen at bifurcation of endograft: .... HU
Iliac lumen at Distal end of right limb of endograft .... HU
Iliac lumen at distal end of left limb of endograft .... HU

Phase 10 Seconds

Image quality: Fully diagnostic 5 4 3 2 1 Non-diagnostic

Endoleak seen: Yes/No

Details:	Endoleak 1	Endoleak 2	Endoleak 3
Type:	Ia/Ib/II/III/Other	Ia/Ib/II/III/Other	Ia/Ib/II/III/Other
Inflow point(s):			
HU at inflow:			
Outflow point(s):			
HU at outflow:			
Certain of flow direction:	Yes/No	Yes/No	Yes/No

Hounsfield Unit measurements: (excluding any calcification of graft structures)

Inferior vena cava: .... HU
Aortic lumen at superior fabric markers of endograft: .... HU
Aortic lumen at bifurcation of endograft: .... HU
Iliac lumen at Distal end of right limb of endograft .... HU
Iliac lumen at distal end of left limb of endograft .... HU

For peer review only - http://bmjopen.bmj.com/site/about/guidelines.xhtml

For peer review only

Phase 15 Seconds

Image quality: Fully diagnostic 5 4 3 2 1 Non-diagnostic

Endoleak seen: Yes/No

Details:	Endoleak 1	Endoleak 2	Endoleak 3
Type:	Ia/Ib/II/III/Other	Ia/Ib/II/III/Other	Ia/Ib/II/III/Other
Inflow point(s):			
HU at inflow:			
Outflow point(s):			
HU at outflow:			
Certain of flow direction:	Yes/No	Yes/No	Yes/No

Hounsfield Unit measurements: (excluding any calcification of graft structures)

Inferior vena cava: .... HU
Aortic lumen at superior fabric markers of endograft: .... HU
Aortic lumen at bifurcation of endograft: .... HU
Iliac lumen at Distal end of right limb of endograft .... HU
Iliac lumen at distal end of left limb of endograft .... HU

Phase 20 Seconds

Image quality: Fully diagnostic 5 4 3 2 1 Non-diagnostic

Endoleak seen: Yes/No

Details:	Endoleak 1	Endoleak 2	Endoleak 3
Type:	Ia/Ib/II/III/Other	Ia/Ib/II/III/Other	Ia/Ib/II/III/Other
Inflow point(s):			
HU at inflow:			
Outflow point(s):			
HU at outflow:			
Certain of flow direction:	Yes/No	Yes/No	Yes/No

Hounsfield Unit measurements: (excluding any calcification of graft structures)

Inferior vena cava: .... HU
Aortic lumen at superior fabric markers of endograft: .... HU
Aortic lumen at bifurcation of endograft: .... HU
Iliac lumen at Distal end of right limb of endograft .... HU
Iliac lumen at distal end of left limb of endograft .... HU

For peer review only - http://bmjopen.bmj.com/site/about/guidelines.xhtml

For peer review only

Phase 25 Seconds

Image quality:	Fully diagnostic	5	4	3	2	1	Non-diagnostic

Endoleak seen: Yes/No

Details:	Endoleak 1	Endoleak 2	Endoleak 3
Type:	Ia/Ib/II/III/Other	Ia/Ib/II/III/Other	Ia/Ib/II/III/Other
Inflow point(s):			
HU at inflow:			
Outflow point(s):			
HU at outflow:			
Certain of flow direction:	Yes/No	Yes/No	Yes/No

Hounsfield Unit measurements: (excluding any calcification of graft structures)

Inferior vena cava: .... HU
Aortic lumen at superior fabric markers of endograft: .... HU
Aortic lumen at bifurcation of endograft: .... HU
Iliac lumen at Distal end of right limb of endograft .... HU
Iliac lumen at distal end of left limb of endograft .... HU

Venous Phase

Image quality:	Fully diagnostic	5	4	3	2	1	Non-diagnostic

Endoleak seen: Yes/No

Details:	Endoleak 1	Endoleak 2	Endoleak 3
Type:	Ia/Ib/II/III/Other	Ia/Ib/II/III/Other	Ia/Ib/II/III/Other
Inflow point(s):			
HU at inflow:			
Outflow point(s):			
HU at outflow:			
Certain of flow direction:	Yes/No	Yes/No	Yes/No

Hounsfield Unit measurements: (excluding any calcification of graft structures)

Inferior vena cava: .... HU
Aortic lumen at superior fabric markers of endograft: .... HU
Aortic lumen at bifurcation of endograft: .... HU
Iliac lumen at Distal end of right limb of endograft .... HU
Iliac lumen at distal end of left limb of endograft .... HU

Other comments / diagnostic findings:

For peer review only - http://bmjopen.bmj.com/site/about/guidelines.xhtml

Section & Topic	No	Item
**TITLE OR ABSTRACT**		
	1	Identification as a study of diagnostic accuracy using at least one measure of accuracy (such as sensitivity, specificity, predictive values, or AUC)
**ABSTRACT**		
	2	Structured summary of study design, methods, results, and conclusions (for specific guidance, see STARD for Abstracts)
**INTRODUCTION**		
	3	Scientific and clinical background, including the intended use and clinical role of the index test
	4	Study objectives and hypotheses
**METHODS**		
Study design	5	Whether data collection was planned before the index test and reference standard were performed (prospective study) or after (retrospective study)
Participants	6	Eligibility criteria
	7	On what basis potentially eligible participants were identified (such as symptoms, results from previous tests, inclusion in registry)
	8	Where and when potentially eligible participants were identified (setting, location and dates)
	9	Whether participants formed a consecutive, random or convenience series
Test methods	10a	Index test, in sufficient detail to allow replication
	10b	Reference standard, in sufficient detail to allow replication
	11	Rationale for choosing the reference standard (if alternatives exist)
	12a	Definition of and rationale for test positivity cut-offs or result categories of the index test, distinguishing pre-specified from exploratory
	12b	Definition of and rationale for test positivity cut-offs or result categories of the reference standard, distinguishing pre-specified from exploratory
	13a	Whether clinical information and reference standard results were available to the performers/readers of the index test
	13b	Whether clinical information and index test results were available to the assessors of the reference standard
Analysis	14	Methods for estimating or comparing measures of diagnostic accuracy
	15	How indeterminate index test or reference standard results were handled
	16	How missing data on the index test and reference standard were handled
	17	Any analyses of variability in diagnostic accuracy, distinguishing pre-specified from exploratory
	18	Intended sample size and how it was determined
**RESULTS**		
Participants	19	Flow of participants, using a diagram
	20	Baseline demographic and clinical characteristics of participants
	21a	Distribution of severity of disease in those with the target condition
	21b	Distribution of alternative diagnoses in those without the target condition
	22	Time interval and any clinical interventions between index test and reference standard
Test results	23	Cross tabulation of the index test results (or their distribution) by the results of the reference standard
	24	Estimates of diagnostic accuracy and their precision (such as 95% confidence intervals)
	25	Any adverse events from performing the index test or the reference standard
**DISCUSSION**		
	26	Study limitations, including sources of potential bias, statistical uncertainty, and generalisability
	27	Implications for practice, including the intended use and clinical role of the index test
**OTHER INFORMATION**		
	28	Registration number and name of registry
	29	Where the full study protocol can be accessed
	30	Sources of funding and other support; role of funders

For peer review only - http://bmjopen.bmj.com/site/about/guidelines.xhtml
